# What Are High Prices

**Published:** 1881-09

**Authors:** 


### WHAT ARE HIGH PRICES.

Items of Interest for June contains Dr. Younger's bill of $435.00 for dental operations. The publicity you give and your comments indicate you regard the bill as unreasonable. Did all dentists charge like Dr. Younger, dental fees would be unreasonable. But no more unreasonable than were all to charge the low price some dentists charge. There is no doubt many thoroughly competant dentists who work for fifty cents per hour. Others charge $2.00 to $5.00 per hour. Some others, like Drs. Younger and Atkinson, charge $10 to $20 per hour. We know the world is apt to form strange judgments about the compensation men should receive for their services, and it very often happens that intellects of the highest order, and hands the best trained, receive the poorest compensation for their labor. Take 1000 dentists, and we are certain they make less clear money than 1000 men of equal ability in any calling in life.

The biggest fortune we ever heard of a dentist making in California was $50,000 in five years. In the same time the most successful one in twenty other occupations we could name, had made from $200,000 to $2,000,000. History does not record a single instance of a dentist becoming rich from the proceeds of his practice. Dr. Younger's bill, that he had manhood enough to go into court and collect, taught the world a good lesson, namely, a dentist's time and advice has value. San Francisco has about 150 dentists. Only 25 of these have an income of $5,000 per annum; the next 25 about $2,500 per annum. The remaining 150 live on faith and chance. Yet the popular impression is that dentists have big incomes, when the plain truth is, fewer of them than of any other class of equal intelligence, make a competence. The dentist who works for the deserving poor for 50cts. an hour, merits the good will of the profession and the gratitude of the public. But if that same dentist works for business men that are making from $10,000 to $500,000, he does right in charging so that he can make as much as some of them he works for. Suppose Dr. Younger's income is from $10,000 to $20,000. Plenty of men with less ability than he, in other pursuits, are making in some cases ten times as much. When a dentist presents his card to his patrons and says his fee is $5, $10, or $20 per hour, he is doing nothing but what is right and honest in making them pay that fee, and the dentist who refuses to work for men who are making $10,000 to $200,000 per year unless he can earn as much as some of them, benefits the profession and merits its gratitude.—(*Items of Interest.*) CALIFORNIA.

